# Genetic markers and continuity of healthy metabolic status: Tehran cardio-metabolic genetic study (TCGS)

**DOI:** 10.1038/s41598-020-70627-5

**Published:** 2020-08-12

**Authors:** Omid Gharooi Ahangar, Niloufar Javanrouh, Maryam S. Daneshpour, Maryam Barzin, Majid Valizadeh, Fereidoun Azizi, Farhad Hosseinpanah

**Affiliations:** 1 Obesity Research Center, Research Institute for Endocrine Sciences, Shahid Beheshti University of Medical Science, P.O. Box: 19395-4763, Tehran, Iran; 2 Cellular and Molecular Endocrine Research Center, Research Institute for Endocrine Sciences, Shahid Beheshti University of Medical Sciences, Tehran, Iran; 3 Endocrine Research Center, Research Institute for Endocrine Sciences, Shahid Beheshti University of Medical Science, Tehran, Iran

**Keywords:** Genetics, Obesity

## Abstract

Obese individuals can be categorized as “healthy obese” (MHO) and “unhealthy obese” (MUO) based on the presence or absence of metabolic abnormality. This study sets out to assess potential genetic causes behind persistence of healthy metabolic status in individuals categorized as “healthy obese”. This study was conducted in the framework of the Tehran cardio-metabolic genetic study (TCGS). 766 MHO subjects at the start of the study followed up 15 years for occurrence of metabolic unhealthy status. These two groups (persistent MHO, MUO) were compared regarding the presence or absence of 16 single nucleotide polymorphisms (SNPs) identified as being associated with obesity phenotype in previous studies. We used logistic regression model for assessing the association between MHO/MUO with candidate SNPs. By the end of the follow up, 206 (27%) were categorized as the persistent MHO and 560 (73%) as MUO groups. Considering interaction effect between some SNP and sex, a sex stratification analysis was applied. When the analysis was performed by gender, rs1121980 associated with a decrease, and rs7903146 with an increase in the likelihood of persistent MHO individuals. Another analysis was separately performed on postmenopausal women from both groups; it showed that rs13107325 was associated with an increase in the likelihood of persistent MHO status in this subgroup of woman. In all cases, the markers had dominant inheritance. This findings suggest that the expression of some genetic markers are associated with persistence of healthy metabolic status, in female obese individuals.

## Introduction

Obesity and overweight have a clear association with long-term morbidity and mortality^1,2^. There is a wide range of heterogeneity among obese individuals with the combination of obesity and metabolic status causing a wide spectrum of obesity phenotypes^3,4^. Prevalence of “healthy obese” is reported to be between 9 and 34% in different populations depending on various definitions of metabolic healthy status^5–8^.

Whether “healthy obese” has a benign course is controversial and different studies have reported varying results^9–12^. Some studies have shown that this obesity phenotype is a dynamic entity and in cohort studies up to 50% of individuals who were “healthy obese” turned into “unhealthy obese” at the end of the follow-up period^5–8^.

Some studies have assessed the predictors of changes in metabolic health in healthy obese individuals. Achilike et al.^13^ suggested that lipid profile and insulin resistance predict the metabolic status in obesity. Appleton et al.^6^ reported that younger age and lower waist circumference (WC) at baseline of obese individuals were significantly associated with maintaining the metabolic healthy state in a longitudinal follow-up. In addition, a study on Tehranian healthy abdominal obese individuals showed that baseline hypertriglyceridemia, low HDL-C, and HOMA-IR were independent predictors of metabolic changes in this population^5^. However, it has been known that obesity and the resulting metabolic abnormality are complex traits that are affected by genetic and environmental factors.

Although genome-wide association studies (GWAS) have identified prevalent genetic variants with small effects, larger steps and more studies are required that identify rare variants with larger effect sizes^14–16^. To the best of our knowledge, few studies specifically focused on assessing the association between single nucleotide polymorphisms (SNPs) and obesity phenotypes^17–24^. However, most of them have cross-sectional design which is not appropriate to derive causal relationships. In this regard, there is a paucity of cohort studies assessing the natural course of healthy obesity. On the other hand, few identified SNPs have been reported in Iranian populations and there is little commonality between the SNPs identified in these studies. This phenomenon could be due to genetic differences between various ethnicities. Iran is a wide country with various weather and religions. The choice of spouse in Iran is highly influenced by religion and place of residence. Because of this, the prevalence of consanguineous marriage is high. This leads to genetic similarities in non-familial individuals in addition to the environmental factors^25^.

This study sets out to assess potential genetic causes behind persistence of healthy metabolic status in individuals categorized as “healthy obese”.

## Materials and methods

### Subjects

This study was conducted in the framework of the Tehran Cardio-metabolic Genetic Study (TCGS) whose genetic information is available^26^. Briefly, the TCGS is part of an ongoing family-based cohort study, the Tehran lipid and glucose study (TLGS)^27^ since 1999, in which subjects have been genotyped and followed up for cardio-metabolic risk factors every 3 years. For this study, 13,426 subjects selected from phases I and II and followed until the end of phase V (2014). Study participants consisted of all subjects who had the following criteria: age ≥ 18 years, body mass index (BMI) ≥ 25 kg/m^2^, metabolic health information being recorded in at least 3 out of 5 phases of the TLGS, healthy metabolic state at the start of the study while being overweight or obese throughout the study. In addition, individuals with a history of steroid use or pregnancy were excluded from the study.

Written informed consent was obtained from each subjects. All procedures in this research were in accordance with the Helsinki declaration and the study protocol was approved by the ethical committee of the Research Institute for Endocrine Sciences, Shahid Beheshti University of Medical Sciences, Tehran, Iran. Any techniques of this study were performed in accordance with the ethical standards, then confirmed by cellular and molecular endocrine research center and obesity research center.

### Methods

At each visit, all subjects interviewed private session. All subjects signed a written consent which referred to Lab and training physicians for clinical and blood sampling. The collected data included medication use, family history of diabetes, and age. The level of physical activity was assessed by The Lipid Research Clinic (LRC) questionnaire in the TLGS first phase. Considering the lack of accuracy in the LRC results, the activity of the subjects in the follow-up was measured in leisure time, job, and home management tasks using the Modifiable Activity Questionnaire (MAQ). Since the LRC does not measure the duration of physical activity, the first TLGS participants attending minimum three weekly sessions of vigorous physical activity were considered physically active. Subjects achieving at least 600 MET.minute/week examined in the second TLGS follow-up were defined as physically active. A digital scale accurate to the nearest 100 g was used to weigh the subjects in light clothing and bare feet. A tape meter was used to measure the subjects’ height in a standing position, with normal-state shoulders and bare feet. The weight in kilograms divided by the height square in meters was used as the formula to calculate the body mass index. The subjects’ waist circumference at the umbilicus level was measured by a non-stretch tape meter accurate to the nearest 0.1 cm, while no pressure was applied to the body surface. After 15 min of rest, the blood pressure was measured using a mercury sphygmomanometer. The subjects were asked to sit and their systolic (SBP) and diastolic (DBP) blood pressure levels with a 30-s interval were measured in order to determine the peak inflation level; to determine a blood pressure, the mean of SBP and DBP was calculated for each subject. Blood parameters were also studies; to obtain blood samples at the TLGS research laboratory, the subjects were asked to fast overnight for 12 h. Using glucose oxidase, the enzymatic colorimetric method was employed to measure fasting plasma glucose (FPG). The triglyceride (TG) and total cholesterol (TC) serum levels were measured to determine the subjects’ lipid profile; for this purpose, according to the manufacturer’s instructions, cholesterol esterase and cholesterol oxidase, and glycerol phosphate oxidase were respectively added to the reactions in the specific kits relied on the enzymatic colorimetric method (Pars Azmoun, Tehran, Iran). The serum high-density lipoprotein level was measured using phosphotingstic acid. The measurement of all samples in the current study met the acceptance criteria according to the internal quality control. In the current study, 0.6% and 1.6 for TG, both 2.2% for serum glucose, and 0.5% and 2 for HDL were the intra- and inter-assay coefficients of variation at baseline. The enzyme-linked immune sorbent assay was employed to determine the insulin level (Mercodia, Sweden), which were 1.4% and 3.3 for intra- and inter-assay coefficients of variation, respectively^27^. Homeostatic model assessment-insulin resistance (HOMA-IR) ¼ [fasting insulin (mU/mL) _ fasting glucose (mmol/L)]/22.5 was used as a formula to determine insulin resistance (IR)^28^.

For assessing SNPs, first the samples were washed with lysis buffer where PBS and RBCs were separated out. Then, through alkaline boiling method, DNA was extracted from the WBCs and the cell extracts were stored at − 20 °C. Quantitative and qualitative assessments on the extracted DNA were performed by electrophoresis and spectrophotometry. Genomic samples were assayed by Human OmniExpress-24-v1-0 (Illumina Inc., San Diego, CA) chip for genotyping marker identification^26^.

### Definition of terms

Based on the inclusion criteria, participants entering in phases I and II of TCGS were divided into “healthy obese” and “unhealthy obese” groups. The “healthy obese” were individuals with constant metabolic health throughout the study. Unhealthy obese was defined as the metabolically healthy subjects at the study baseline diagnosed in at least one phase of the TLGS with metabolic syndrome (MetS), which persisted until the end of the study. To MetS was defined in the study using the following criteria for joint interim statement^29^: (1) Hypertension as DBP ≥ 85 and SBP ≥ 130 mmHg, or use of antihypertensive agents; (2) fasting HDL < 40 mg/dL (1.03 mmol/l) and < 50 mg/dL (1.29 mmol/L) in males and females respectively, or medication use; (3) TG ≥ 150 mg/dL (1.7 mmol/L) or medication use; and (4) FPG ≥ 100 mg/dL (5.6 mmol/L), two-hour blood glucose ≥ 140 mg/dL (7.8 mmol/L), or medication use. The national WC cutoff points (≥ 91 and ≥ 89 cm for females and males, respectively) were used to define abdominal obesity. Individuals with BMI ≥ 25 kg/m^2^ and a maximum of one criterion out of the 5 criteria were considered “persistent metabolic healthy obese” (MHO) and those with BMI ≥ 25 kg/m^2^ and at least 2 criteria considered as “metabolically unhealthy obese” (MUO). IR was defined according to the study by Tohidi et al., as HOMA-IR ≥ 2.6 for both genders^30^.

### Study protocol

**Figure 1:**
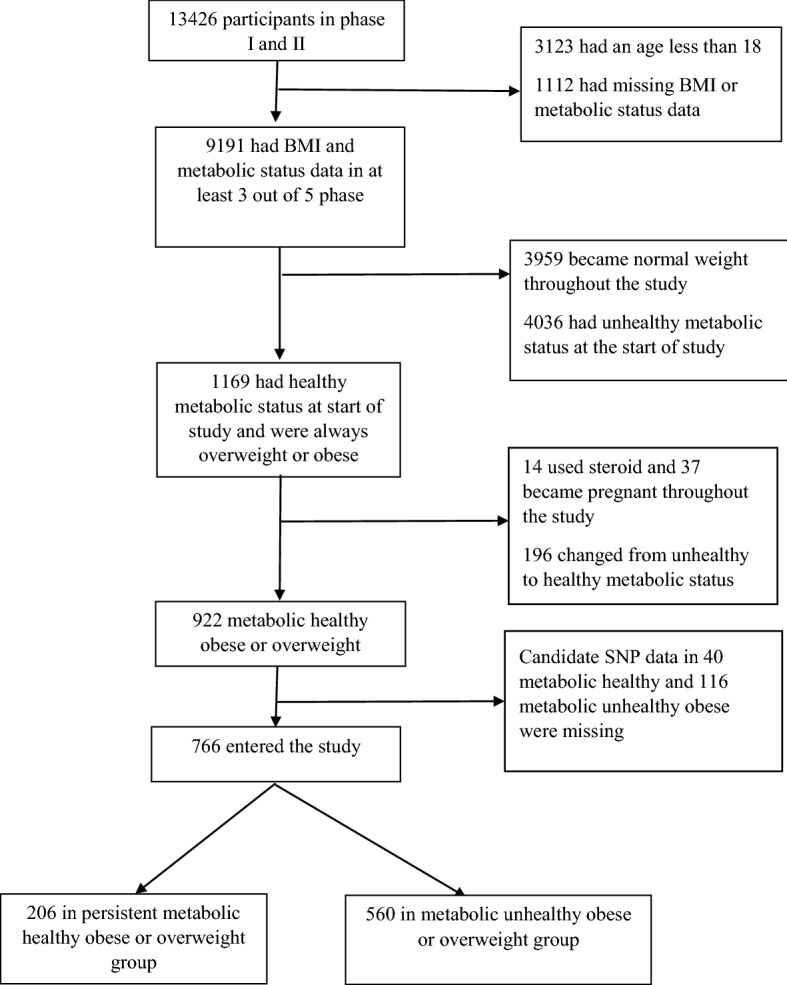
Flow diagram of study.

These two groups were compared regarding the presence or absence of 17 single nucleotide polymorphisms (SNPs) identified as being associated with obesity phenotype in previous studies. For identifying the 16 SNPs, all relevant published studies were assessed and the SNPs with a statistically significant association with metabolically unhealthy status were collected^17–24^. Out of the 54 SNPs selected, the information on 16 SNPs was available in TCGS data (including rs569356, rs3790433, rs1514175, rs560887, rs16858082, rs13107325, rs1799883, rs7903146, rs2237892, rs2237897, rs756534, rs6539019, rs1121980, rs2287019, rs6098242, rs223750) and used in the present study (Supplementary Table 1) . Figure 1 shows the flow diagram of the study.

### Statistical method

Data for continuous variable are expressed as mean ± standard deviation (SD), and categorical variables are expressed as percentages. Independent *t*-test and chi-square test were used for continuous and categorical variable, respectively. Furthermore, we used logistic regression model for assessing the association between MHO/MUO with candidate SNPs considering age, age power 2, gender and first two principal components as covariates. Further, Kaplan Meier and Log Rank statistics were used to compare the age free of MUO between men and women with obesity. We used PLINK^31^ and R^32^ programs for descriptive and data analyses. All the analyses were considered at 0.05 level of significance.

## Results

Of the total 13,426 participants in the Tehran Cardio-metabolic Genetic Study (TCGS), after considering the inclusion and exclusion criteria, 766 individuals finally entered the cohort. By the end of the follow up, 206 (27%) were categorized as the persistent MHO and 560 (73%) as MUO groups.

**Figure 2:**
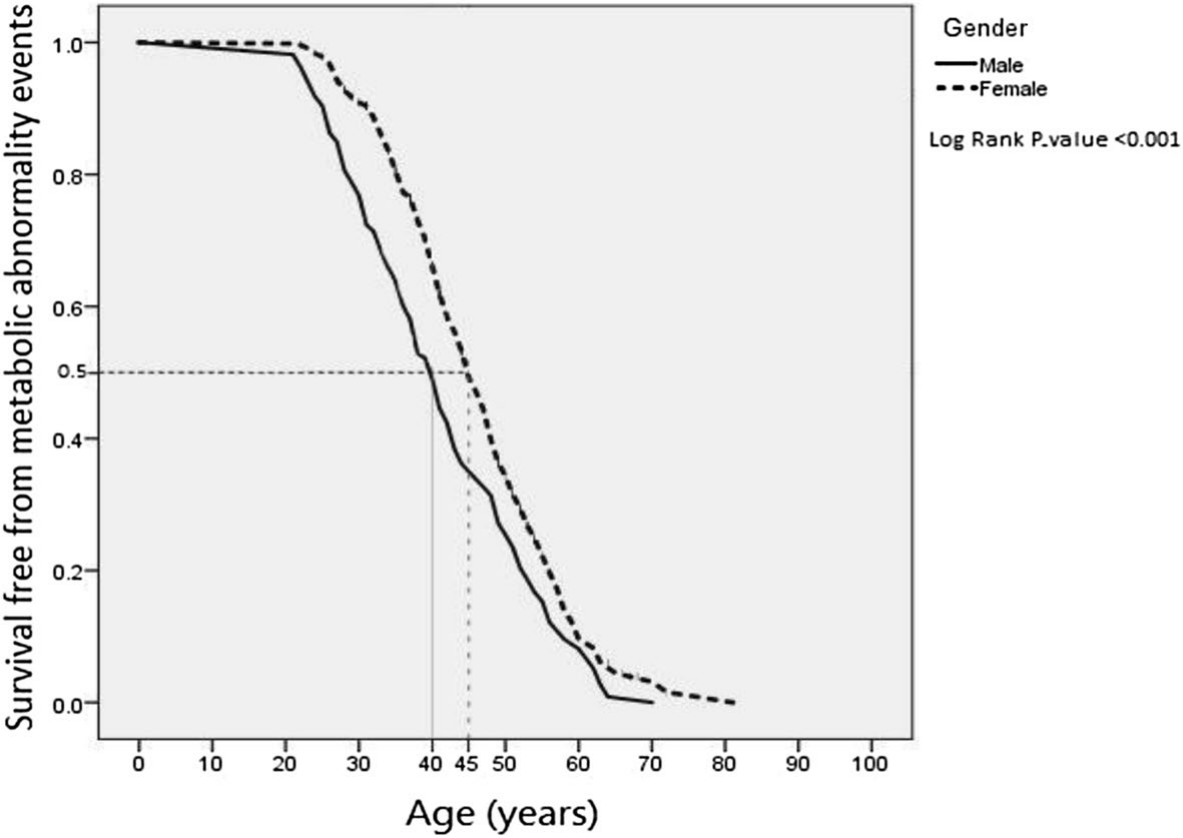
Kaplan–Meier curves for cumulative survival free from metabolic abnormality events.

The half of females and males were free from metabolic abnormalities at age 40 CI (38.2–41.8) and 46 CI (45.0–46.9), respectively (Fig. 2). As can be seen, the survival curves differed significantly from each other (log rank test, *P* < 0.001). The baseline characteristics of 766 individuals when entering the study (phase I or II) are shown in Table 1. The mean age in MHO and MUO groups were 30 and 35 years, respectively. A total of 169 subjects (82%) of the MHO and 378 (67.5%) of the MUO group were women which where statistically significant (*P* < 0.001). There were significant differences in all baseline characteristics between the two groups (*P* < 0.05), except for physical activity level, homeostatic model assessment of HOMA-IR and IR. Thus, these baseline variables were more favorable in the persistent MHO group compared to the MUO. At the end of the follow up, when evaluating the metabolic status of female subjects, 30 (17.8%) and 78 (20.6%) of the persistent MHO and MUO groups were in menopause respectively, which was not a statistically significant difference between the two groups (*P* = 0.06).

**Table 1:** Baseline characteristics of the 766 individuals of Tehran cardio-metabolic genetic study with 15 years follow up.

Variable*	MHO			MUO			P-value**		
	Female	Male	Total	Female	Male	Total	Female	Male	Total
Participant, n (%)	169 (82)	37 (18)	206	378 (67.5)	182 (32.5)	560	< 0.001	< 0.001	ND
Age (year)	30 ± 8	27 ± 8	29 ± 8	36 ± 10	34 ± 11	35 ± 10	< 0.001	0.02	< 0.001
FPG (mg/dl)	84 ± 7	86 ± 6	85 ± 7	87 ± 11	88 ± 6	87 ± 9	< 0.001	NS	< 0.001
SBP (mmHg)	105 ± 9	108 ± 7	106 ± 8	111 ± 11	112 ± 14	111 ± 12	< 0.001	NS	< 0.001
DBP (mmHg)	71 ± 7	69 ± 8	70 ± 7	74 ± 7	73 ± 9	74 ± 8	< 0.001	NS	< 0.001
TG (mg/dl)	86 ± 32	97 ± 33	88 ± 32	110 ± 42	124 ± 57	115 ± 51	< 0.001	NS	< 0.001
HDL (mg/dl)	51 ± 11	45 ± 6	50 ± 10	47 ± 11	43 ± 8	46 ± 10	< 0.001	NS	< 0.001
WC (cm)	80 ± 7	90 ± 9	82 ± 8	86 ± 7	89 ± 7	87 ± 7	< 0.001	NS	< 0.001
BMI (kg/m^2^)	27 ± 2	27 ± 3	27 ± 2	28 ± 2	27 ± 2	28 ± 2	< 0.001	NS	< 0.001
HOMA-IR	1.6 ± 0.77	1.9 ± 0.8	1.7 ± 0.78	1.8 ± 1.01	1.79 ± 1.00	1.8 ± 1.00	0.04	NS	NS
IR, n (%)	10 (5.9)	5 (13.5)	15 (7.3)	44 (11.6)	11 (6.0)	55 (9.8)	NS	NS	NS
Low physical activity (METS)	28 (16.6)	4 (10.8)	32 (15.5)	75 (19.8)	37 (20.3)	112 (20)	NS	NS	NS
Non-smoker, n (%)	161 (95.3)	30 (81)	191(92.7)	357 (94.4)	137 (75.3)	494 (92.2)	NS	0.02	0.02
Menopause, n (%)	30 (17.8)		ND	78 (20.6)		ND	NS	ND	ND

*MHO* metabolic healthy obese; *MUO* metabolic unhealthy obese; *FPG* fasting plasma glucose; *SBP* systolic blood pressure; *DBP* diastolic blood pressure; *TG* triglyceride; *HDL* high density lipoprotein; *WC* waist circumference; *BMI* body mass index; *HOMA-IR* homeostatic model assessment of insulin resistance; *IR*, insulin resistance; *NS* not significant at 0.05 level; *ND*, not done.

^*^Variables expressed as means ± standard deviation or number of individuals (percentages).

^**^P-value related to comparing MHO and MUO groups using t-test / Chi Square test for quantitative / qualitative variables, respectively.

To determine the relationship between candidate genetic markers and persistence of MHO individuals, the logistic regression with dominant model was used. Comparing the two groups, when all participants including both men and women entered the model, none of the markers showed any association. Considering significant interaction effect between some SNP and sex (*P* for interaction < 0.001), a sex stratification analysis was applied. When the analysis was performed by gender, rs1121980 associated with a decrease, and rs7903146 with an increase in the likelihood of persistent MHO individuals. Furthermore, another analysis was separately performed on postmenopausal women from both groups (*P* for interaction < 0.001 for menopausal status); it showed that rs13107325 was significantly different between the two groups. It was associated with an increase in the likelihood of persistent MHO status in this subgroup of woman. In all cases, the markers had dominant inheritance, suggesting that having an allele of the intended genes can increase or decrease the likelihood of persistence of MHO status. Information on the effective genetic biomarkers in the cohort study is summarized in Tables 2 and 3.

**Table 2:** Association between candidate genetic markers and continuity of healthy metabolic status in woman of Tehran cardio-metabolic genetic study with 15 years follow up.

Sample	Gene	Chr	Marker	Base pair	Major/minor	OR	95% CI	P-value	MAF
Female	FTO	16	rs1121980	53,775,335	G/A	2.12	(1.03–2.75)	0.03	0.39
Female	TCF7L2	10	rs7903146	112,998,590	C/T	0.22	(0.05–0.93)	0.04	0.35
Menopause	SLC39A8	4	rs13107325	102,267,552	C/T	0.27	(0.07–0.97)	0.04	0.04
Non menopause	SLC39A8	4	rs13107325	102,267,552	C/T	0.89	(0.56–1.4)	0.62	0.04

*MAF* minor allele frequency, *Chr* chromosome, *OR* odd ratio.

**Table 3:** Association between candidate genetic markers and continuity of healthy metabolic status in men of Tehran cardio-metabolic genetic study with 15 years follow up.

Sample	Gene	Chr	Marker	Base pair	Major/minor	OR	95% CI	P-value	MAF
Men	SLC39A8	4	rs13107325	102,267,552	C/T	3.50	(0.44–27.43)	0.23	0.04
Men	FTO	16	rs1121980	53,775,335	G/A	0.79	(0.37–1.65)	0.53	0.39
Men	TCF7L2	10	rs7903146	112,998,590	C/T	0.73	(0.35–1.54)	0.41	0.35

*MAF* minor allele frequency, *Chr* chromosome.

In addition, a cross-sectional statistical analysis at the end of the study was performed based on the metabolic status of participants in phase V of the TCGS (1,772 and 5,455 in the MHO and MUO groups, respectively), which demonstrated no significant association between the candidate genetic markers and metabolic status of participants.

## Discussion

The current prospective cohort study was performed on 766 individuals from the TCGS cohort, and the association between 16 genetic biomarkers (which were related to MetS in previous investigations) and persistence of MHO status was assessed.

In the course of 15 years of follow up, 560 out of 766 participants (73%) changed from healthy metabolic state to unhealthy. This percent change was greater than in previous studies^5–8^. This difference is because of various metabolic healthy state definitions and different lengths of follow up.

Previous studies have evaluated different predictors of changes in metabolic health in healthy obese individuals. Some indicators of MetS (WC, hypertriglyceridemia, low HDL-C at baseline) and individual features such as younger age are have been reported as independent predictors of unhealthy status^5,6,33^. Comparing the two groups in our study, the healthy obese group had a younger age as well as lower BMI and rate of smoking, physical inactivity, and IR. This difference was not significant regarding physical activity and IR, due to the large number of missing data (25 and 40%, respectively). The data on other individual characteristics such as diet, alcohol use, and socio-economic status were very diverse and could be misjudged. Although MetS indices were within the normal range in both groups, these variables were more favorable in the MHO group. Further, the two groups were significantly different in the percentage of females, with a higher percentage of women being in the MHO group as compared to the MUO (82% vs 67.5%); thus, an analysis based on gender was separately performed for each group.

Many studies assessed the role of different genetic markers in development of unhealthy metabolic status in healthy obese subject. After the statistical analysis, three genes including SLC39A8, FTO, and TCF7L2 on chromosomes 4, 16 and 10, respectively, were associated with the metabolic status in participants. The FTO gene has been identified as an effective factor on appetite and satiety^34^; in the HUNT study, it has been related to WC and TG level^17^. Also, in our study, it was associated with increased risk of MetS. The TCF7L2 is related to insulin secretion^18^; although in some previous studies^19^ it has been associated with an increase in the risk of MetS, it had a protective effect for the persistence MHO in our study. This difference may be due to different minor allele frequencies in our study (MAF 0.35 Vs 0.21), study designs (cohort vs cross-sectional), definitions of MetS, and study population.

The SLC39A8 gene, from the solute-carrier family, plays a role in zinc transport. Considering the proposed roles of zinc in pancreatic β cells, it is not far from expectation that zinc transporters such as the product of SLC39A8 gene would play a part in insulin secretion^35^. In the current study, this gene had a protective effect on preservation of individuals’ metabolic health.

Eftekharzade et al.^36^ found that among all suggested predictors of metabolic outcome, female gender played the strongest role. Also, in our study all genes were significant only in women. Initially, it was assumed that this finding may be due to the effect of estrogen on the metabolism of pre-menopause women^37^. Thus, the percentage of menopause women was compared between the two groups, which had no significant difference (17.8% vs 20.6%). Also, a separate analysis was performed between menopause women of the two groups, showing protective effect of the SLC39A8 gene in the menopause female population (Table 2).

Io the best of our knowledge, very few studies have investigated the effect of gender on the expression of genes associated with MetS. For example, in the HUNT study^17^, the FTO gene was related to increased weight only in women; this gender-dependent effect of the FTO gene on sensitivity to insulin and serum glucose level has been observed in children as well^38^. This association also applies to other SNPs. For example, Schlauch et al.^39^ showed an association between 89 SNP with cardio-metabolic disease, only in obese woman. Rarely, some investigations have reported an association between obesity and markers on the X chromosome. Among genes, member 14 of SLC6A (located on q arms of X chromosome) affects the appetite control and body weight by changes in serotonin synthesis and serotonergic receptor mechanisms^40^. Although an association between persistence of metabolic health and the 8th member of the SLC39 family was detected in our study, their association is questionable. This female gender-dependent relation has not been studied for other markers. Meanwhile, in other study of Sedaghati-Khayat et al.^41^, with was similar to our study population, they could not show any association between FTO gene and metabolic healthy obesity, probably due to disregarding the female effect and study design. Thus, it is hoped that if future studies are conducted with a good design and focus on the effect of gender, we could gain more favorable results. Also, we can develop prediction models such as Choe et al.^42^ in Korea, for metabolic status in obese or overweight woman in our population, if dietary and alcohols consumption data are included.

The current study is one of the first investigations in our country and the few in the world with a cohort design and appropriate follow-up period which was done specifically on overweight or obese individuals. As with other studies, a cross-sectional analysis was performed at the end of the study (phase V of the TCGS) based on metabolic status of subjects, with 7,227 participants (1,772 and 5,455 in the MHO and MUO groups, respectively), where no significant association was found between the markers and metabolic status of individuals. This confirms that cross-sectional studies are inefficient in showing this association. Further, this study is one of the few investigations indicating genetic markers being dependent on the female gender, and suggesting its possible influence on the expression of genetic markers which are effective in MetS. However, the greatest limitation of the present study was the low number of participants, which was inevitable due to the inclusion and exclusion criteria. Moreover, greater female to male ratio could in part explain the lack of significant association between SNPs and healthy status in males. However, inadequate power in male subjects cannot invalid our findings in females. Also, due to the insufficient number of participants, individuals with overweight were also included in the study. Last but not least, lack of data on diet, alcohol use, and socio-economic status were other limitations of the current study.

## Conclusion

The present study indicated that the FTO (rs1121980) and TCF7L2 (rs7903146) genes on chromosomes 16 and 10 respectively had a significant association with persistence of healthy metabolic status in healthy obese. Both of the genes were only significant in women. Furthermore, SLC39A8 (rs13107325) on chromosome 4 had a significant association with healthy metabolic status in menopausal women.

Of these three genetic markers, two (rs13107325 and rs7903146) had a protective effect on the preservation of metabolic health, while the other marker (rs1121980) was associated with an increased risk of MetS. The findings of our study suggest that the expression of these genetic markers, which are related to MetS in obese individuals, has an association with the female gender. However, we believe that; due to in adequate power in males; lack of associations cannot be strongly concluded. Confirmation of this hypothesis requires further investigation.

## Supplementary information

Supplementary Information
