# Supplementary material for: Genetic markers and continuity of healthy metabolic status: Tehran cardio-metabolic genetic study (TCGS)
Source: Sci Rep. 2020 Aug 12;10:13600. doi: 10.1038/s41598-020-70627-5 (PMC7423921; doi:10.1038/s41598-020-70627-5)
Supplement: Supplementary file 1 — Supplementary Information [file 41598_2020_70627_MOESM1_ESM.pdf]

Title:

Genetic markers and continuity of healthy metabolic status: Tehran Cardio-metabolic Genetic Study (TCGS)

Authors:

Omid Gharooi Ahangar (MD)[1], Niloufar Javanrouh (PhD) [2], Maryam Daneshpour (PhD)[2*], Maryam Barzin (MD, PhD) [1], Majid Valizadeh (MD)[1], Fereidoun Azizi (MD)[3], Farhad Hosseinpanah (MD)[1*].

Affiliations:

[1] Obesity Research Center, Research Institute for Endocrine Sciences, Shahid Beheshti University of Medical Science, Tehran, Iran

[2] Cellular and Molecular Endocrine, Research Center, Research Institute for, Endocrine Sciences, Shahid Beheshti, University of Medical Sciences, Tehran, Iran

[3] Endocrine Research Center, Research Institute for Endocrine Sciences, Shahid Beheshti University of Medical Science, Tehran, Iran

*** Correspondences:**

1-Farhad Hosseinpanah, MD.
Obesity Research Center
Research Institute for Endocrine Sciences
Shahid Beheshti University of Medical Sciences, Tehran, Iran
P.O. Box: 19395-4763
Phone: +98-21-22432500
Cell: +98-912-343 1927
Fax: +98-21-22416264
Email: fhospanah@endocrine.ac.ir

2-Maryam S. Daneshpour, PhD.
Cellular and Molecular Endocrine Research Center,
Research Institute for Endocrine Sciences
Shahid Beheshti University of Medical Sciences, Tehran, Iran.
Email: daneshpour@sbmu.ac.ir

Supplementary: Table 1- The description of 16 identified SNPs

**SNP**	**Chr: Position**	**Reference Allele**	**MAF**	**Consequence**	**Gene**
rs1121980	16:53775335	A	0.39	intron	FTO
rs13107325	4:102267552	A	0.04	missense	SLC39A8
rs1514175	1:74525960	G	0.45	intron	TNNI3K
rs16858082	4:45173787	C	0.34	intergenic	-
rs1799883	4:119320747	A	0.32	missense	FABP2
rs223750	X:89603383	G	0.12	intergenic	-
rs2237892	11:2818521	T	0.03	intron	KCNQ1
rs2237897	11:2837316	T	0.03	intron	KCNQ1
rs2287019	19:45698914	T	0.15	intron	QPCTL
rs3790433	1:65428659	G	0.32	intron	LEPR
rs560887	2:168906638	C	0.22	intron	G6PC2
rs569356	1:28810174	G	0.07	upstream_gene	OPRD1
rs6098242	20:54793941	A	0.22	intergenic	-
rs6539019	12:101909308	G	0.49	intron	DRAM1
rs756534	12:101886477	T	0.41	intron	DRAM1
rs7903146	10:112998590	G	0.35	intron	TCF7L2

SNP, single nucleotide polymorphisms; Chr, chromosome; MAF, minor allele frequency.
